# Identification and Detection of *Botryosphaeria dothidea* from Kiwifruit (*Actinidia chinensis*) in China

**DOI:** 10.3390/plants10020401

**Published:** 2021-02-20

**Authors:** Li Wang, Hui Hou, Zengqiang Zhou, Hongtao Tu, Hongbo Yuan

**Affiliations:** Zhengzhou Fruit Research Institute, Chinese Academy of Agricultural Sciences, Zhengzhou 450009, China; wangli06@caas.cn (L.W.); houhui@caas.cn (H.H.); zhouzengqiang@caas.cn (Z.Z.)

**Keywords:** kiwifruit ripe rot, *Botryosphaeria dothidea*, identification, LAMP, detection

## Abstract

Kiwifruit is very popular among consumers due to its high nutritional value. The increasing expansion in kiwifruit cultivation has led to the spread of rot diseases. To identify the pathogens causing kiwifruit ripe rots in China, 24 isolates were isolated from the diseased fruit and wart in trees. *Botryosphaeria dothidea* was recognized as the pathogen causing kiwifruit ripe rot and wart in the tree through internal transcribed spacer (ITS) sequencing, pathogenicity testing, morphological and microscopic characteristics. The rapid and accurate detection of this pathogen will lead to better disease monitoring and control efforts. A loop-mediated isothermal amplification (LAMP) method was then developed to rapidly and specifically identify *B. dothidea*. These results offer value to further research into kiwifruit ripe rot, such as disease prediction, pathogen rapid detection, and effective disease control.

## 1. Introduction

Kiwifruit (*Actinidia chinensis*) is a deciduous tree belonging to the Actinidiaceae family. Kiwifruit has been popular due to its balanced nutritional minerals, dietary fibers, high vitamin C contents, and health-promoting metabolites [1]. At present, it is widely planted in China, New Zealand, Italy, Iran, and so on [2,3].

The climate, environment, and mode of production changes have led to the spread of various diseases including rots, which can accelerate the softening rate, shorten the storage period and seriously affect the quality and taste of kiwifruit [4]. So far, several fungi including *Alternaria*, *Colletotrichum*, *Botryosphearia*, *Cylindrocarpon*, *Phomopsis*, *Phoma*, *Diaporthe*, *Botrytis*, and *Penicillium* have been reported in association with kiwifruit rot [5,6,7,8,9,10,11]. *Botryosphaeria dothidea*, frequently isolated from rotted fruit, is one of the major causing agents of post-harvest rot of kiwifruit, an important disease of *A. deliciosa* [2]. *B. dothidea* is a fungal pathogen capable of infecting a wide range of woody plant species, and causes different disease phenotypes including fruit rot, dieback, stem canker, and tree death [12]. Clear external symptoms of ripe rot were sometimes absent from the surface of the fruit, but a portion of the fruit surface becomes collapsed. If the collapsed portion of the fruit is peeled off, the sunken area shows the appearance of water-soaked flesh tissue. The milky-colored spot, which is present in the flesh, develops concentrically as the fruit ripens [13]. However, it is difficult to distinguish it from the other rot kiwifruit based on symptoms.

Thus, accurate and rapid approaches for the detection of *B. dothidea* are essential to rapidly and effectively control the spread of rot diseases and to guide appropriate fungicide selection. Traditional pathogen identification approaches are based upon morphological properties such as pathogen size, shape, color, conidial texture, colony morphology, and cell wall thickness [14,15]. The identification of pathogens based upon these phenotypic traits, however, can often be inaccurate. For example, *B. dothidea* exhibits phenotypic traits that are similar to those of related *Botryosphaeriaceae* species [12]. Besides, such phenotypic characterization is a labor-intensive process that necessitates isolating and preparing pure cultures from individual fungal isolates [16].

Many researchers have developed DNA-based approaches for the detection and identification of pathogens on a molecular basis. Among them, PCR-based assays are the most commonly used approaches [16]. These strategies generally take advantage of species-specific differences in pathogen ribosomal DNA (rDNA) internal transcribed spacer (ITS), tubulin, and elongation factor-1a sequences to design primers capable of identifying specific pathogens of interest [12,17]. PCR-based assays have previously been leveraged to detect *Botryosphaeriaceae* species associated with a range of different hosts [18]. While these approaches are simpler and less time-intensive than traditional approaches, they are expensive, and require complicated instrumentations such that they are difficult to be utilized in the field.

Loop-mediated isothermal amplification (LAMP) is a novel DNA amplification strategy that proceeds at a constant temperature and is highly accurate and specific while allowing for naked eye visualization and analysis of the LAMP reaction products eye via incorporation of metal-ion-based indicators or DNA-intercalating dyes [19,20]. LAMP does not require a thermal cycler, electrophoresis, or gel imaging system, thus, is promising and is used widely for detecting a range of pathogens at field levels [21,22]. LAMP assay has been used to detect the *B. dothidea* causing Chinese hickory canker and apple ring rot [23,24], but only detected the pathogen in trunk. Whether a similar approach can be used to diagnose kiwifruit ripe rot caused by *B. dothidea*, however, remains to be determined.

The aim of the study was to identify and detect of *B. dothidea* causing ripe rots of kiwifruit in China. As such, the first objective of this study was to identify the pathogen causing kiwifruit ripe rots in China via a combined analysis of microscopic, morphological, and molecular characteristics, and pathogenicity testing. The second objective was to develop a LAMP-based approach to specifically detect *B. dothidea* isolate. Together, the results of this study offer value to further research into kiwifruit ripe rot, such as disease prediction, pathogen rapid detection, and effective disease control.

## 2. Results

### 2.1. Isolate Characterization

**Figure 1:**
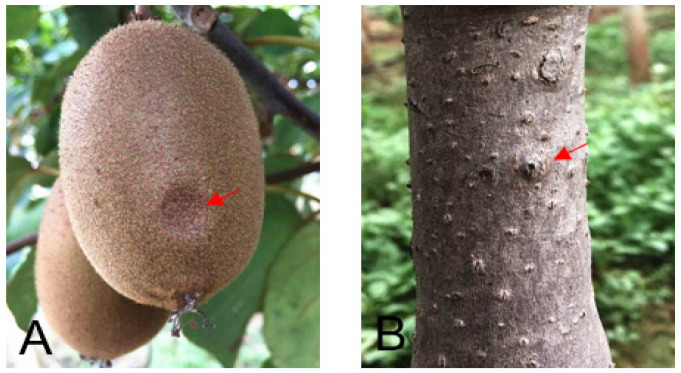
Symptoms of kiwifruit soft rot. (**A**) External slightly sunken symptom (arrow) on kiwi fruit. (**B**) Wart (arrow) on kiwi stem.

**Figure 2:**
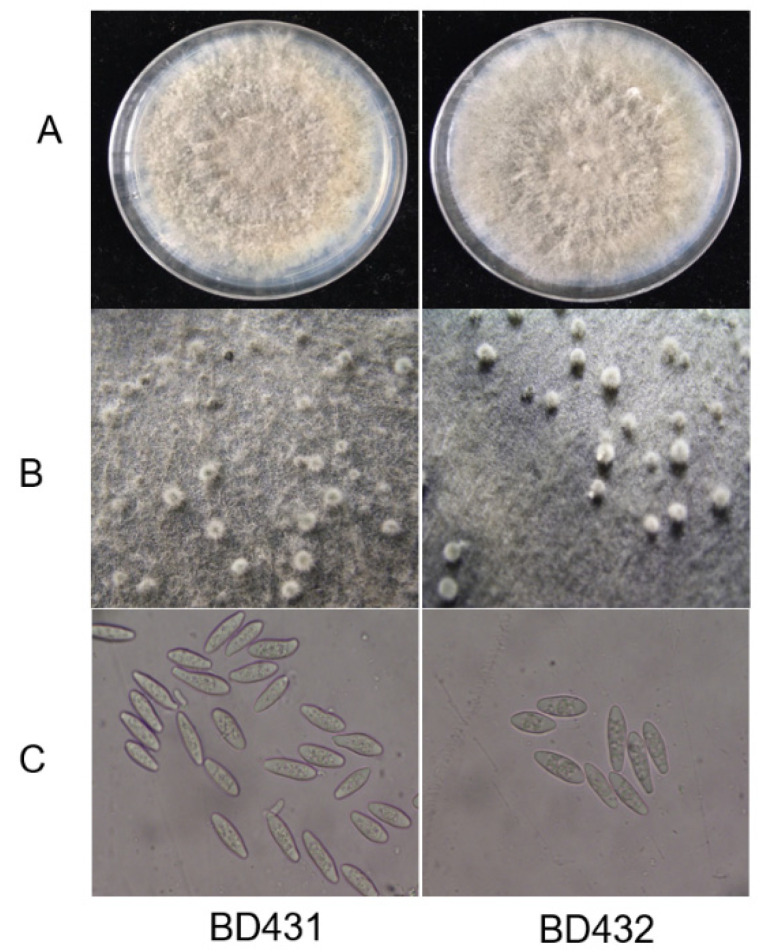
Morphologic characteristics of *Botryosphaeria dothidea*. Bd 431 comes from the diseased fruit (**left**), Bd432 comes from the wart (**right**). (**A**) The *B. dothidea* phenotype following a 5-day culture period on potato dextrose agar (PDA). (**B**) Pycnidia formation of *B. dothidea* on PDA. (**C**) Conidial morphology of *B. dothidea*.

Naturally diseased fruits were healthy in appearance, with soft tissues and slightly sunken infected sites (Figure 1A). Infected branches or trunks of trees had warts (Figure 1B). Isolates (24) were isolated from all the samples, 12 isolates (include Bd431) come from the diseased fruit, while 12 isolates (include Bd432) come from the wart. On potato dextrose agar (PDA), initially greenish-brown to gray colonies were formed by all isolates, which were then turned into dark gray after the incubation of five days (Figure 2A). After nine days, the isolates started to produce pycnidia (Figure 2B). Conidia were hyaline, unicellular, and fusoid, the size of the conidia of isolates producing abundant spores was (21.2–27.5) × (5.2–7.1) µm (Figure 2C). Based on morphological characters, the fungus matched the holotype strains CBS 115476 of *B. dothidea* (Moug. ex Fr.) Cesati and De Notaris [15].

### 2.2. Molecular Identification

**Figure 3:**
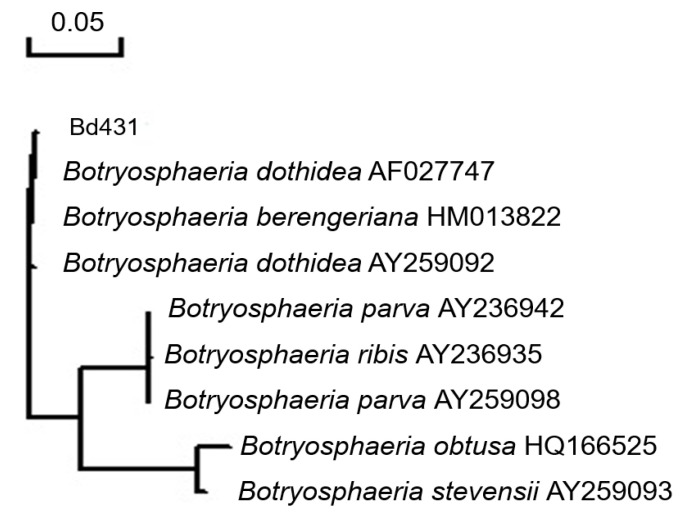
Dendrogram constructed by the neighbor-joining method showing the phylogenetic relationship (100%) between the isolate of *Botryosphaeria dothidea* (BD431) and *B. dothidea* isolate (AF027747) based on the We have checked (ITS) sequences.

The use of ITS1/ITS4 primers caused the amplification of the expected bands in Bd431 and Bd432 isolates, which was in accordance with the classification of the morphological features employed in this research. For BD431 isolate, the region sequence of ITS rDNA revealed 100% homology to *B. dothidea* isolate (AF027747) (Figure 3), and the sequence was deposited in Genbank with accession number MW547773. The β-tubulin gene and the EF1α gene were successfully amplified for the isolate, the β-tubulin sequence analysis of the isolate revealed 100% homology to *B. dothidea* isolate (KY393166), and the EF1α gene also has 100% homology to *B. dothidea* isolate (GU294736). Moreover, the BD432 isolate has the same result. The results of ITS and β-tubulin and EF1α identification were found to be identical with analysis of microscopic and morphological characteristics.

### 2.3. Pathogenicity Testing

**Figure 4:**
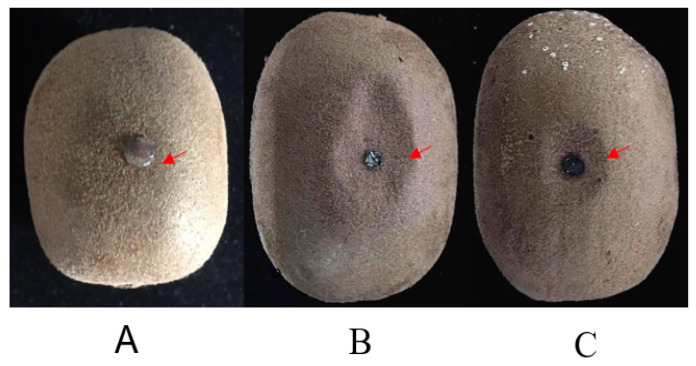
Symptoms of kiwifruit inoculated by *Botryosphaeria dothidea*. (**A**) No external symptom (arrow) of kiwifruit artificial infection with fungi-free PDA. (**B**) External slightly sunken symptom (arrow) of kiwifruit artificial infection with *B. dothidea* (Bd 431 comes from the diseased fruit). (**C**) External slightly sunken symptom (arrow) of kiwifruit artificial infection with *B. dothidea* (Bd432 comes from the wart).

In pathogenicity assays, disease symptoms appeared as soft lesions with slightly sunken infected sites (Figure 4). On the other hand, all the non-inoculated control fruits remained undecayed.

### 2.4. Reisolation and Identification of Infected Fungi

From the lesion margins, the decayed tissue samples were shifted to PDA plates, reisolated the fungus, and identified as the inoculated strain by analyzing the microscopic, morphological, and molecular characteristics.

### 2.5. LAMP Primers Selection

**Figure 5:**
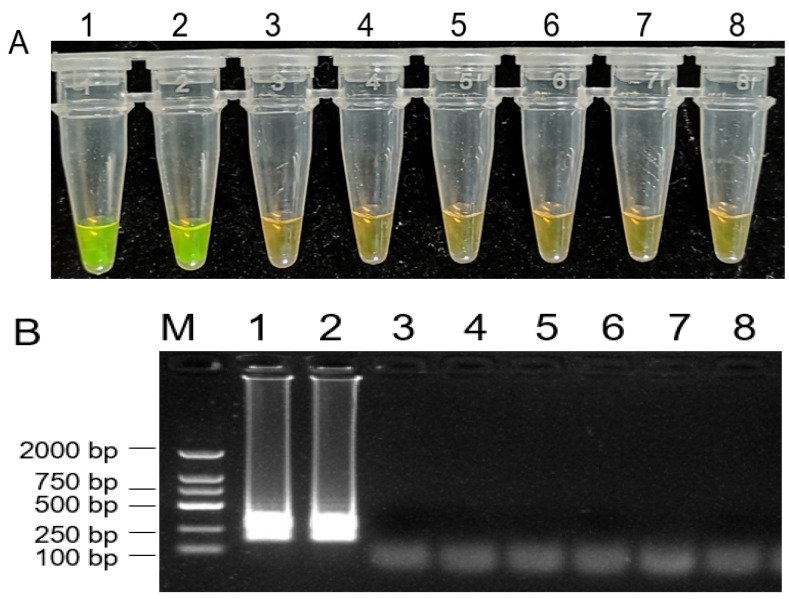
Loop-mediated isothermal amplification (LAMP) assay on detection of *Botryosphaeria dothidea*. 1000 × SYBR Green I color change-based LAMP product detection (**A**). Agarose gel electrophoresis-based LAMP product detection (**B**). M: DNA marker 2 k, 1: *Botryosphaeria dothidea* isolate from kiwifruit fruit, 2: *Botryosphaeria dothidea* isolate from kiwifruit stem, 3: *Botrytis cinerea*, 4: *Colletotrichum gloeosporioides*, 5: *Valsa mali*, 6: *Alternaria alternate*, 7: *Marssonina coronaria*, 8: water.

To design the specific primers for the detection of *B. dothidea* species, ITS sequences of *B. dothidea* and several other fungi were used for alignment analysis. Based on the diversity of the ITS regions, we designed a set of four primers for LAMP assays to amplify the specific ITS fragment of *B. dothidea*. Through a series of adjustments and optimization of reaction conditions, a successful LAMP assay for the detection of *B. dothidea* was developed in this study. As shown in Figure 5A, only the reactions containing DNA from *B. dothidea* isolates displayed positive reactions as indicated by a visible fluorescent yellow-green color, whereas the samples containing DNA from other fungi isolates were negative reactions with orange color, and a ladder-like electrophoresis pattern (Figure 5B).

### 2.6. Assessment of LAMP Assay Sensitivity

**Figure 6:**
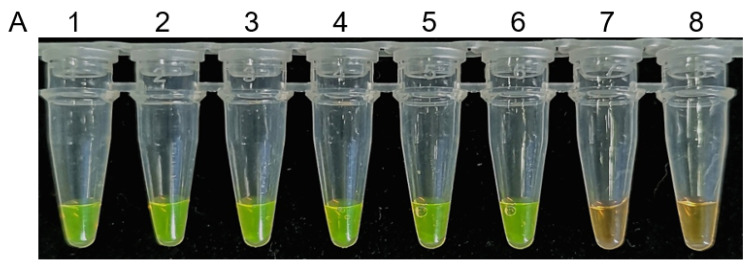

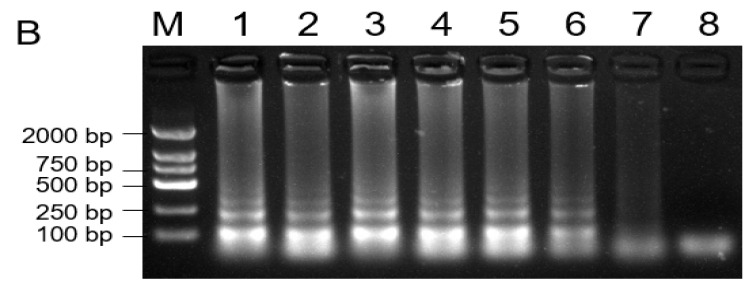
Assessment of LAMP assay sensitivity for the detection of *Botryosphaeria dothidea.* 1000 × SYBR Green I color change-based LAMP product detection (**A**). Agarose gel electrophoresis-based LAMP product detection (**B**). DNA isolated from *Botryosphaeria dothidea* isolate was subjected to serial 10-fold dilution (10 ng–10^−6^ ng) and served as a template in LAMP assays. M: DNA marker 2 k, 1–7: 10 ng, 10^−1^ ng, 10^−2^ ng, 10^−3^ ng, 10^−4^ ng, 10^−5^ ng, 10^−6^ ng, 8: water.

The sensitivity of this LAMP assay was determined by using DNA serial 10-fold dilutions of DNA from a *B. dothidea* isolate (Bd-432) and was subjected to serial 10-fold dilution (10 ng to 10^−6^ ng). This assay has the potential to detect as low as 10^−5^ ng of DNA, with clear color changes and DNA ladder products consistent with a positive reaction (Figure 6), demonstrating high sensitivity of the assay for the detection of *B. dothidea* isolates.

### 2.7. LAMP-Mediated Detection of B. dothidea in Fruit Samples

**Figure 7:**
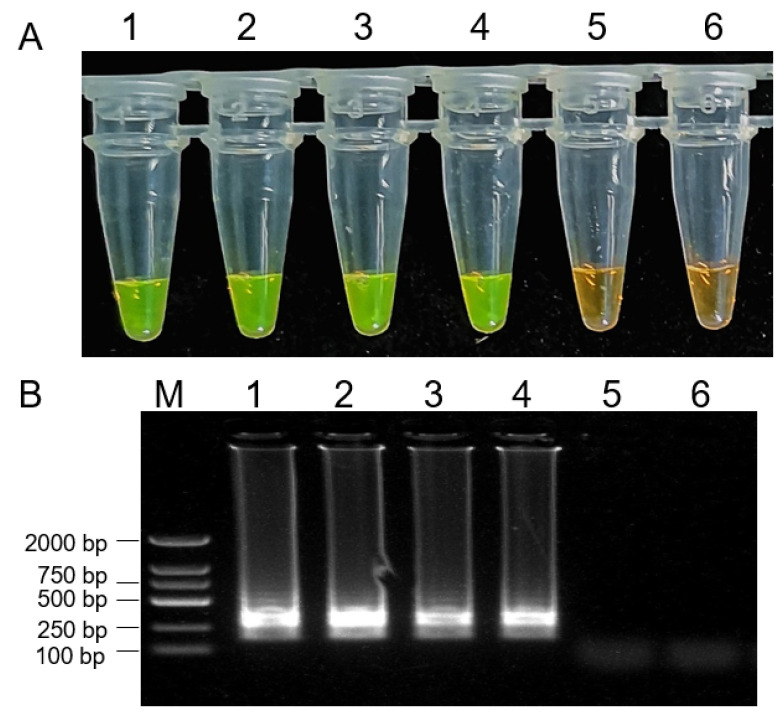
Detection of the *Botryosphaeria dothidea* in tissue samples from diseased kiwi fruits via LAMP assay. 1000 × SYBR Green I color change-based LAMP product detection (**A**). Agarose gel electrophoresis-based LAMP product detection (**B**). M: DNA marker 2 k, 1: DNA from *Botryosphaeria dothidea* isolate mycelium, 2: DNA from diseased kiwi fruit infected with *Botryosphaeria dothidea* from the pathogenicity determination experiment, 3,4: DNA from diseased kiwi fruit infected with *Botryosphaeria dothidea* from field, 5: DNA from healthy kiwifruit fruit, 6: water.

To assess the ability of LAMP assay for the detection of *B. dothidea* isolates in diseased apple tissues, samples were collected from healthy kiwifruit and kiwifruit infected with *B. dothidea* isolates. Samples from kiwifruit infected with *B. dothidea* isolates yielded positive LAMP reactions (Figure 7). Moreover, we isolated the diseased kiwifruit fruits from the field for the LAMP assay. Based on the morphology and biological characters, the isolates were identified as *B. dothidea*. These findings confirmed that our LAMP assay could be successfully used to rapidly and accurately diagnose kiwifruit rot caused by *B. dothidea* strain isolates.

## 3. Discussion

Previous studies revealed that various pathogens are not only able to cause the infection but also responsible for the severe deterioration of trees and harvested fruit quality during the cold- or post-storage ripening [25]. *B. dothidea* caused ripe rot affects harvested fruits during post-storage ripening. While during cold storage, *Botrytis cinerea* based *Botrytis* storage rot affects harvested fruits [26]. *Sclerotinia* rot, caused by *Sclerotinia sclerotiorum*, mainly affects immature fruits on the trees [27,28]. In this paper, the symptom of infected rot fruit on the trees conformed to “ripe rots”; the observed symptoms were similar as reported previously from Iran, China, Korea, and New Zealand [1,8,9,12]. Although there were many reports about fruit ripe rot caused by *B. dothidea*, few reports exist about stem symptoms. In this study, fruit showing rot symptoms and warts on the stem were collected from kiwifruit orchards. The results of the study suggested that *B. dothidea* was the pathogen that causes the kiwifruit ripe rots and stems warts in China. No other pathogens were isolated in this research, probably because the sample size is not enough.

Approaches to treating and controlling this disease remain limited, and a novel approach to effectively and reliably detecting *B. dothidea* must be developed. A range of molecular techniques has been designed in recent years to detect plant pathogens including bacteria, viruses, oomycetes, and fungi [29,30,31,32,33]. Several strategies including PCR-based approach, restriction fragment length polymorphism (RFLP), and high-resolution melting have been used to detect isolates. While these approaches are simpler and less time-intensive than traditional approaches, they are expensive and require complicated instrumentations such that they are difficult to be utilized in the field.

LAMP assays represent a novel, efficient, specific, sensitive, and low-cost DNA amplification approach that is highly amenable to application in the field as it does not require expensive equipment [19]. Such LAMP assays have previously been employed successfully to detect Chinese hickory canker and apple ring rot caused by *B. dothidea* [23,24]. In this study, we designed a LAMP assay that was able to detect *B. dothidea*. Our results confirm the value of our newly developed LAMP assay as a highly sensitive method of detecting *B. dothidea* isolates with a DNA detection limit of 10–5 ng. This LAMP sensitivity threshold is consistent with reported thresholds in other pathogen detection analyses [17,34]. Notably, our LAMP assay was 100-fold more sensitive than a previously reported quantitative LAMP (q-LAMP) assay designed to detect Chinese hickory canker caused by *B. dothidea* [23]. This LAMP assay can also be employed to identify *B. dothidea* within samples of diseased fruit, thus, offering clear value as a test that can be conducted in the field for *B. dothidea* detection. This approach, thereby, allows for the dynamic monitoring of *B. dothidea* spread, enabling farmers to select appropriate fungicides to optimally protect their crops.

## 4. Materials and Methods

### 4.1. Isolates Used and Morphological Observation

Twelve kiwifruit fruits showing rot symptoms and twelve warts on branches (important cultivars: *A. chinensis* var. chinensis “Jinyan”) were collected from orchards of a main kiwifruit-producing area, Xixia County (111°01′ E 33°05′ N), in 2019, which is a temperate continental monsoon climate; the climate is mild, the sunshine is sufficient in the southwest of Henan Province in China. The pathogens were isolated by the tissue isolation method. They were disinfected with 0.5% NaClO for 1 min and washed twice with sterile distilled water. Small pieces from the edges of diseased tissue were placed on potato dextrose agar (PDA) (200 g potato extracts L-1, 2% glucose, and 2% agar) medium. After culturing for five days at 25 °C, a mycelial plug was collected from the growing edge of each colony and transferred to a new PDA plate followed by incubation at 25 °C for seven days. Emerging colonies were transferred several times by the hyphal tipmethod until and pure cultures were obtained [35].

After five days, the morphological features of the culture of each isolate were studied on PDA at 25 °C. Malt extract agar (MEA, 2%) was used to culture the isolates by applying 12 h of an intermittent cycle of near black UV light (2U, Cnlight, Nanjing, China) to induce sporulation [36].

### 4.2. DNA Extraction, Sequencing, and Analysis

Isolates of Bd431 and Bd432 were grown on PDA for five days and mycelium was used to extract the genomic DNA by following the CTAB method described by Möller et al. [37]. DNA concentration of each sample was quantified by using a NanDrop UV spectrophotometer (NanoVue Plus, GE Healthcare Life Sciences, New York, NY, USA). Phylogenetic analyses of ITS, translation elongation factor 1-α, and beta-tubulin were used to identify *B. dothidea* [38], so the three genes were used in our study. Primers ITS1 and ITS4 were used to amplify the ITS region of DNA [39], while amplification of partial β-tubulin gene was accomplished by the primers Bt2a and Bt2b [40], and for the partial elongation factor 1α gene with the primers EF1-728F and EF1-986R [41].

The polymerase chain reaction (PCR) was performed in an iCycler Thermal Cycler (Bio-Rad Laboratories Inc., Hercules, CA, USA) in a final volume of 25 μL containing 12.5 μL of 2 × PCR mix (Sangon Biotech Co., Ltd., Shanghai, China), 1.5 μL of each primer (10 μM), 1 μL of DNA (10 ng), and 8.5 μL of double-distilled H_2_O. The PCR conditions include the 3 min duration for the initial denaturation at 94 °C; followed by 35 cycles of 30 s at 94 °C, 30 s at 55 °C, and 90 s at 72 °C; and a final extension of 10 min at 72 °C. The resulting PCR products were analyzed by gel electrophoresis in a 1% agarose gel in 1 × Tris-borate-EDTA buffer and sequenced by Sangon Biotech Co., Ltd. (Shanghai, China). Amplified sequence was compared with the other sequences found in the National Center for Biotechnological Information (NCBI) database (http://www.ncbi.nlm.nih.gov, accessed on 20 February 2021) using Basic Local Alignment Search Tool (BLAST) [42].

### 4.3. Pathogenicity Testing

The selected isolates were screened for their pathogenicity on kiwifruits according to their morphological and molecular data. The pathogenicity of the two isolates was assessed by growing these isolates on kiwifruit fruits under laboratory conditions. Briefly, ‘Jintao’ kiwifruits were rinsed and disinfected for 5 min via treatment with 1% NaOCl, after which they were washed two times using sterile dH_2_O and dried in a transfer hood. From each isolate, mycelial plugs with a diameter of 5 mm were obtained from the edges of five-day-old colonies and added to the surface of each kiwifruit, with control apples instead being inoculated using fungi-free PDA. Three replicates per treatment condition were prepared on three different apples. Following inoculation, fruits were transferred to a sterilized plastic box and were incubated at 25 °C. This analysis was repeated two times.

### 4.4. Reisolation and Identification of Infected Fungi

To complete Koch’s postulates, fungi were isolated from diseased parts of inoculated fruit [43]. Surface disinfection of lesion edges was performed in 0.5% NaOCl for 60 s followed by three times washing with sterile-distilled water and then kept on PDA in dishes for seven days at 25 °C. Identification of fungi was based on conidial morphology and the sequence of the rDNA ITS region as described previously.

### 4.5. LAMP Primers Design for the Detection of Botryosphaeria dothidea

The obtained ITS sequence was compared with that another four different fungal isolates including *Botrytis cinerea*, *Colletotrichum gloeosporioides*, *Valsa mali*, *Alternaria alternate*, and *Marssonina coronaria* by multiple sequence alignment using MUSCLE. The design of LAMP primers was prepared according to the sequence variability among the four fungal species. Primer Explorer V4 software was used to design the LAMP primers. We designed four primers (F3: CCGCCAGAGGACCATCAA; B3: CCTTCGGAATACCAAAGGGC; FIP: CCAGAACCAAGAGATCCGTTGTTCTCCAGTCAGTAAACGATGCA; BIP: GATGAAGAACGCAGCGAAATGCCAATGTGCGTTCAAAGATTCGA) as a set of LAMP primers were used to detect the *B. dothidea*. To assess the LAMP primer’s specificity, we also used other samples of fungal species including *Botrytis cinerea*, *Colletotrichum gloeosporioides*, *Valsa mali*, *Alternaria alternata*, and *Marssonina coronaria*. *B. cinerea* was collected from kiwifruit, while other isolates were collected from apples from different orchards in China. All isolates were isolated and identified as described previously. All isolates were cultured on PDA plates at 25 °C for five days, followed by the collection of mycelial samples for genomic DNA extraction, as described previously.

Initial LAMP reaction conditions were selected and modified based on a prior study [44]. Initially, LAMP reactions were carried out in a 25-μL volume, which contained 4 U of a large fragment of Bst DNA polymerase (New England Biolabs), 2.5 μL of 10 × ThermoPol Buffer (New England Biolabs), 4 mM MgSO_4_ (New England Biolabs), 1 mM dNTP, 1.4 μM each FIP and BIP, 0.2 mM each F3 and B3, 0.8 M betaine, and 1 μL target DNA, with ddH_2_O being added to a final volume of 25 μL. As a control, ddH_2_O was instead used. Reaction mixtures were then incubated for 60 min at 60 °C, after which they were heated for 10 min to 80 °C to terminate the reaction via enzymatic inactivation. Next, we added 1 μL of SYBR Green I (1000×) per reaction tube. Positive reactions yielded a fluorescent yellow-green color that was readily detectable to the naked eye, while negative reactions remained brown. The resulting products were analyzed by 1% agarose gel electrophoresis, and experiments were repeated two times.

### 4.6. Assessment of LAMP Assay Sensitivity

The LAMP sensitivity was evaluated by performing serial 10-fold dilutions of template DNA from a *B. dothidea* isolate (Bd-432), with ddH_2_O serving as a negative control. Optimal LAMP reaction parameters, as detailed above, were then used to conduct LAMP detection. The analysis was repeated twice.

### 4.7. LAMP-Mediated Detection of B. dothidea in Fruit Samples

Infected kiwifruit from the pathogenicity determination experiments and different kiwifruit orchards were collected for the test. A piece of tissue was taken from infected kiwifruit and was surface-sterilized with 75% alcohol for DNA extraction. Total genomic gDNA was extracted using a Fast DNA isolation kit (TransGen) based on the provided directions. Then the DNA was used for LAMP assays as above. This experiment was repeated two times.

## 5. Conclusions

In summary, our results provide clear evidence that *B. dothidea* is the pathogen causing kiwifruit ripe rots and stem warts in China. The developed LAMP method represents a rapid, efficient, and effective means of reliably identifying the isolates.

## Data Availability

Not applicable.
